# Older Adults’ Adherence to Technology-Based Intervention: The Role of Messaging and Individual Differences

**DOI:** 10.1093/geroni/igaa057.1821

**Published:** 2020-12-16

**Authors:** Walter Boot, Nelson Roque, Erin Harrell, Neil Charness

**Affiliations:** 1 Florida State University, Tallahassee, Florida, United States; 2 Pennsylvania State University, University Park, Pennsylvania, United States; 3 University of Alabama, Tuscaloosa, Tuscaloosa, Alabama, United States

## Abstract

Adherence to health behaviors is often poor, including adherence to at-home technology-based interventions. This study (N=120) explored adherence to a cognitive training intervention delivered via computer tablet, assessed adherence over a 4.5 month period, explored how individual difference factors shaped adherence, and tested the efficacy of message framing manipulations (positive vs. negative framing) in boosting adherence. Individual difference factors predicted adherence, including variations in self-efficacy and belief in the efficacy of cognitive training. Overall message framing had little impact. However, during the final portion of the study in which participants were asked to play as much or as little as they wanted instead of following a schedule, participants who received positively framed messages engaged with the intervention more. Implications for predicting and boosting adherence to home delivered technology-based interventions will be discussed.

